# Hierarchic Stochastic Modelling Applied to Intracellular Ca^2+^ Signals

**DOI:** 10.1371/journal.pone.0051178

**Published:** 2012-12-27

**Authors:** Gregor Moenke, Martin Falcke, Keven Thurley

**Affiliations:** 1 Mathematical Cell Physiology, Max Delbrück Center for Molecular Medicine, Berlin, Germany; 2 Institute for Theoretical Biology, Charité Universitätsmedizin, Berlin, Germany; Universitat Politecnica de Catalunya, Spain

## Abstract

Important biological processes like cell signalling and gene expression have noisy components and are very complex at the same time. Mathematical analysis of such systems has often been limited to the study of isolated subsystems, or approximations are used that are difficult to justify. Here we extend a recently published method (Thurley and Falcke, PNAS 2011) which is formulated in observable system configurations instead of molecular transitions. This reduces the number of system states by several orders of magnitude and avoids fitting of kinetic parameters. The method is applied to 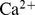 signalling. 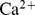 is a ubiquitous second messenger transmitting information by stochastic sequences of concentration spikes, which arise by coupling of subcellular 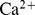 release events (puffs). We derive analytical expressions for a mechanistic 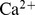 model, based on recent data from live cell imaging, and calculate 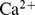 spike statistics in dependence on cellular parameters like stimulus strength or number of 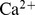 channels. The new approach substantiates a generic 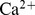 model, which is a very convenient way to simulate 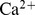 spike sequences with correct spiking statistics.

## Introduction

The molecular state transitions and interactions inside cells forming pathways and functional units are inherently random [1]–[3]. Some cellular subsystems involve sufficiently large molecule numbers to be well described by deterministic mean field dynamics, but many are best described as stochastic processes. Additionally, cells show considerable heterogeneity even within clonal populations. Biological noise and cell-to-cell variability have been identified and studied in chemotaxis [4], gene expression [5], cell signalling [6]–[8] and cell differentiation [9], [10]. A variety of mathematical strategies can be used to analyse these stochastic dynamical systems, including approximation techniques such as Langevin and Fokker-Planck Equations [11], [12], and exact methods like the chemical master equation or its simulation by the Gillespie algorithm [13]. Approximation techniques are often based on the assumption of Gaussian white noise, which is only valid for large copy-numbers of identical components and fluctuations which are small compared to the mean value. However, in biological processes like gene expression [1] or 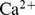 dynamics [14], [15], the change of state of a single molecule may have a huge impact on systems dynamics (see also [16]). To describe such systems without rough approximations, we recently developed a new modelling framework based on emergent behaviour of biomolecules [17]–[19].


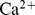
 is a ubiquitous second messenger transmitting information in many cases by repetitive cytosolic concentration spikes [14], [15], [18]. An important class of 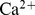 signals is mediated by Inositol-1,4,5-trisphosphate (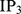 ), which is produced in response to hormonal activation of cell surface receptors [14], [15]. 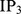 then binds 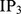 receptors (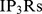 ) in the endoplasmic reticulum (ER) and thus sensitises them for activation by 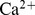. 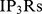 are organised as clusters of about 1 to 20 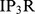 molecules [20]–[22]. Active 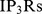 act as 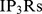 channels, releasing 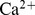 ions from the ER lumen into the cytosol. Upon sensitisation by 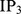 they are successively activated by 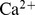 -induced 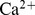 release (CICR). This mechanism is based on the opening probability of 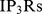, which increases with the local 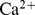 concentration, up to a threshold value where further increase of the 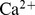 concentration becomes inhibitory [23], [24]. The outflux of 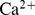 eventually stops either because of depletion of the ER 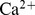 stores or the threshold 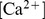 for channel inhibition is reached. Sarco-endoplasmic reticulum 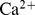 ATPases pump 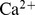 back into the ER after release. The transient increases in cytosolic 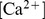 trigger downstream effects like activation of protein kinase C [15], [25]. The clustering of 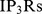 implies that global cell-wide 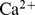 signals result from a hierarchic cascade of single channel opening (‘blips’) over cluster opening (‘puffs’) to opening of several clusters (‘wave’ or ‘spike’), which arise when open clusters open neighbouring clusters due to Ca2+ diffusion and CICR. This cascade is well characterised by live cell imaging and mathematical modelling studies [14], [15]. We and others have recently demonstrated that cellular 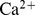 signals are repetitive stochastic events [6], [18], [26], [27], although at certain conditions they appear regular and have long been analysed with deterministic mathematical models [28], [29].

Thus, the 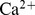 spikes arise by a multiscale stochastic process emerging by clustering of 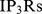. If we seek for a theory to intracellular 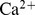 signalling, what are the experimental observations such a theory should explain? 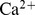 spike sequences are random with average interspike intervals (ISI) from a few tens of seconds to hundreds of seconds. Due to this random spiking, simulation of individual time series of the 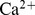 concentration is less meaningful than with deterministic limit cycle oscillators. There are, however, well defined properties of the stochastic sequences characterising the system. The moment relation between the standard deviation 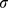 and the average 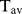 of ISI is linear with a positive slope between 0.2 and 1 [6], the slope of the moment relation of individual cells agrees with the population relation [30], [31], i.e. it is robust against cell-to-cell variability and cell type specific [32]. These results were established by single cell experiments with astrocytes, microglia, PLA stem cells and HEK cells [6]. The ISI obey simple two-parameter distributions [6]. Weakening spatial coupling between 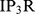 clusters by 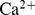 buffers like EGTA destroys cellular spikes and any variation of 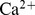 signalling on their time scale [27]. Therefore, a mechanistic theory should be able to explain the emergence of cellular spiking on the basis of cluster properties and cluster coupling only. Moreover, the theory should link the statistical properties to biological parameters like stimulation strength and buffer concentrations.

We can meet all those requirements by an approach called hierarchic stochastic modelling [17]. What is measured *in vivo* can be correctly described only by waiting-time probabilities since it represents transitions between observable states like ‘open’ or ‘closed’ of a specific 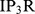 cluster. Hierarchic stochastic modelling deals with waiting-time probabilities for state-changes in a reduced state-space. The reduction of the state space focuses on functional or observational aspects of the system. In contrast the full state space contains all biochemically possible states of the involved molecules. Their dynamics can then be described by rate laws yielding the usual chemical master equation. To avoid computational overhead by computing non observable state transitions and facilitate analytic solutions, all microscopic states corresponding to one function (e.g. for an ion channel ‘open’) are subsumed into one observable state. The set of all observable states forms the reduced state-space. This entails non-exponential waiting-time probabilities instead of rate-laws for the description of transitions between the observable states.

Here, we develop a convenient solution technique for hierarchic stochastic systems by the Laplace transform, which directly leads to analytical expressions for statistical properties of important system variables. Moreover, we present a minimal mechanistic model for stochastic 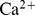 spikes based on recent data from live cell imaging. We provide analytic approximations for the dependence of system statistics on biological parameters. Finally, based on the observation of a very robust time scale separation in the mechanistic model, we find a surprisingly convenient description of 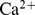 spike statistics, which is in excellent agreement with mechanistic models and with experimental data.

## Results

Cellular 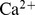 dynamics is a multiscale stochastic process driven by huge gradients in the cytosolic 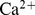 concentration, and detailed computer simulations are difficult to implement and time-consuming [14], [32]. A concise, analytically treatable mechanistic model was published only recently by two of us [17]. In the following, we start by explaining the method that made this possible and presenting an extension to the method, which is solution by Laplace transform of generalised exponential distributions. After that, we construct the input data required for the modelling approach in terms of analytical functions of cellular parameters. The third section analyzes a minimal mechanistic 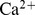 model, which allows for analytical solutions and and elucidates the role of a global negative feedback. Finally, based on that analysis, we present a proper derivation of a very simple description of stochastic 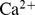 dynamics called generic model, which very nicely describes the experimental 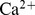 spike statistics.

### Hierarchic Stochastic Modelling Analysed by the Laplace Transform

**Figure 1:**
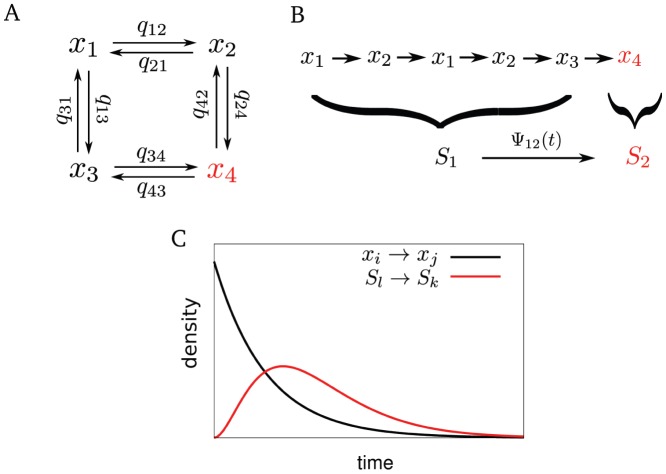
Schematic illustration of hierarchic stochastic modelling. (A) Markovian models consider all possible changes of a molecule 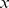, e.g. binding of regulatory factors, production, degradation. Thus, microscopic state transitions 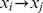 are governed by rate parameters 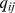. In the case of 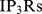, the 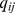 refer to binding (unbinding) of 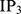 and 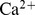 to (of) their respective binding sites on certain 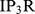 subunits. (B) A typical path of state changes according to (A) includes repetitive switches between individual states before a specific state of interest (here, 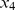) is reached. The observable states 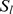 are subsets of all microscopic states (here e.g. 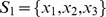). In 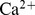 dynamics, observable states may represent open or closed clusters of 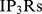. (C) Microscopic transitions 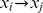 with parameters 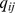 induce exponential waiting time probabilities 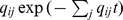. Observable state transitions 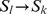 follow waiting time probabilities 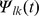, which can have one or several maxima due to their internal dynamics.

The traditional formulation of a stochastic model starts from the state transitions of the 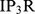 subunits [14]. In most single channel models [33], three binding sites are assumed for each of the four subunits, one activating and one inhibiting site for 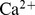 and one site for 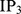. A complete stochastic description determines the time course of the probability for each system state. Their dynamics are described by the master equation [11].
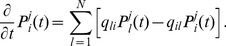
(1)

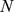
 is the number of system states (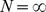 is possible) and 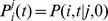 is the conditional probability that the system is in state 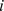 at time 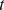 conditioned by being in state 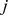 at time 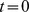. The 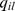 are the rates for state transitions 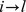 (see Table 1 for a summary of the notation). Figure 1A illustrates the state scheme of a hypothetical receptor channel molecule 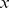 with only four states 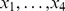 (3 closed states, 1 open state). This case can easily be treated directly by Eq. 1. However, if we consider several interacting molecules with more than 100 possible states each, like those in typical ion channels [33]–[35], Eq. 1 becomes intractable due to the large number of system states.

**Table 1:** Important mathematical symbols used in this work.

Symbol	Meaning	Unit
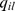	Markovian transition rate from state 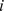 to 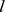	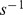
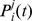	conditional probability 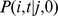	
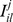	probability flux from state 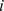 to 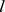 conditioned with 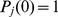	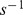
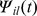	conditioned waiting time to go from 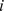 to 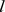	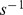
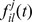	initial function for the Volterra integral equation	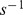
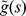	Laplace transform of function 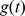	
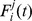	first passage time density (FPT) for the first visit in 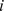 starting in 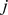	
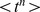	n-th moment of the FPT	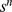
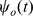	opening probability density	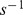
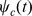	closing probability density	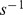
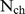	mean number of 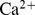 channels involved in a puff event per cluster	
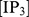	cytosolic 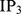 concentration	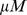
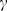	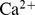 channel closing rate	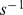
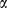	shape parameter of the GE distribution	
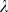	scale parameter of the GE distribution	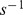
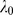	puff rate	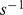
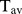	average interspike interval (ISI)	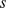
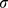	standard deviation of ISIs	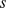
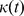	time dependent spike rate	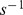
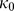	localized spike rate	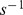
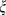	recovery rate of global feedback	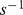
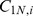	coupling strength of cluster 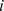	
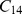	homogeneous coupling strength for the tetrahedron model	
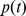	probability density of a spike occurring at epoch 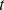	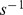

When describing a biological system with a mathematical model, we are often not interested in all the molecular details, but rather in few states that are accessible to experimental quantification or are important for system function. In the case of 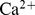 dynamics the main question is whether the channel is closed or not, and not which of the many possible closed states it is in. Nevertheless, the channel state depends on the dynamics between all its closed states, such that a rigorous treatment demands the inclusion of all possible microscopic state changes into Eq. 1. We circumvent state space explosion by lumping microscopic states belonging to the same observable state (Fig. 1B). Consider stochastic transitions between observable system states 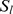, each representing a set of microscopic system states. Because a sequence of microscopic transitions occurs between two consecutive transitions, each transition 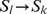 is not governed by a simple rate-law with exponentially distributed waiting-time, as in Eq. 1. The sequence of microscopic transitions causes a non-exponential waiting time distribution (Fig. 1C). These waiting time distributions introduce memory into the process such that the Markov property is lost (see Text S1). The probability densities of the waiting times until specific state transitions 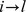 occur, 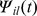, are considered as input data for our 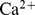 model (see Section Materials and Methods).

For analytical analysis, we need to solve a non-Markovian master equation determined by the 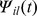. As an extension to the previously published theory [17], we find that analytical solutions for the stationary occupancy probabilities in the observable system states (see Fig. 1) can be obtained by Laplace transform followed by solution of linear algebraic systems (see Section Materials and Methods and Text S1). Importantly, we do not need to calculate the often awkward inverse Laplace transform. We can obtain the average and standard deviation of interevent times which are key characteristics of biological processes like 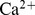 signalling or chemotaxis [17], [36]. The expressions for the moments 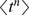 of interevent times are particularly convenient if the system can be represented as a linear chain with 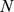 distinct states 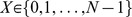 and state transitions 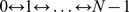 (see Section Materials and Methods). For instance, in the case of 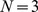 we obtain.
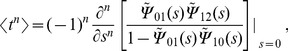
(2)where 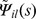 denotes the Laplace transformed conditioned waiting times. From Eq. 2, average 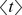 and standard deviation 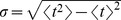 of interevent times are readily computable.

A limiting step in this solution strategy is the availability of the Laplace transform 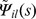. Multistep processes like the ones considered here (see Fig. 1) are often well described by 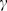-distributions [11], which are also frequently observed in biological dynamical systems [27], [37], [38]. However, expressions like Eq. 2 are impractical for 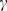-distributed conditioned waiting times. Instead of that, we found that generalised exponential (GE) distributions [39].
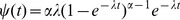
(3)are very convenient for operations in Laplace space and approximate the multistep processes leading to 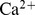 spikes equally well as 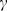-distributions (see below). In Eq. 3, 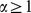 is the shape parameter and 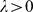 is the scale parameter. Note that for 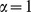, Eq. 3 is an exponential distribution with parameter 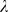.

With the use of Laplace transformed GE distributions, we have found a straight-forward strategy to solve for dynamic properties of non-Markovian systems. Importantly, computational effort is essentially reduced to solution of a linear algebraic system, and thus the strategy can easily be extended to larger systems, in contrast to the Voltera integral equations discussed in Ref. [17].

### Waiting Times of 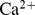 Dynamics and their Dependence on Cellular Parameters

Intracellular 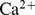 dynamics can be described in terms of an observable open and an observable closed state of clusters of 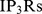 [14], [17], [18]. All microscopic states in which all channels of the cluster are closed belong to the observable closed state. All other states belong to the observable open state. The subunits of individual 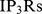 continuously change states by binding and unbinding of regulatory molecules (microscopic dynamics), but only the closing of the last open channel and the first opening when all channels are closed represent transitions between the observable states of a cluster of 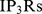 (see Fig. 1). The waiting times until a cluster opens or closes are determined by true probability densities, which we denote by 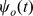 and 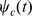, respectively. Cellular 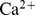 dynamics consist of the action of several interacting clusters, constituting a state space 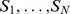 of possible configurations of open and closed clusters. The conditioned waiting times 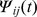 are products of the basic transition probability 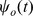 or 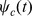 of a cluster and the probabilities that all other clusters remain in their states. 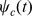 can be derived from experimental data (see Ref. [17] and Text S1 for details). 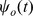 depends on the number of 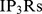 per cluster 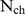 and on the 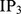 concentration, which represents the strength of a hormonal stimulus in our model. Intracellular 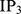 dynamics are still an open question and beyond our scope here. Because of CICR, the individual opening probabilities depend on the local 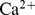 concentration, which is a function of the number of open clusters. The value of the 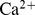 concentration rise caused by an open cluster at the locations of the other clusters gives the strength of spatial coupling between clusters.

**Figure 2:**
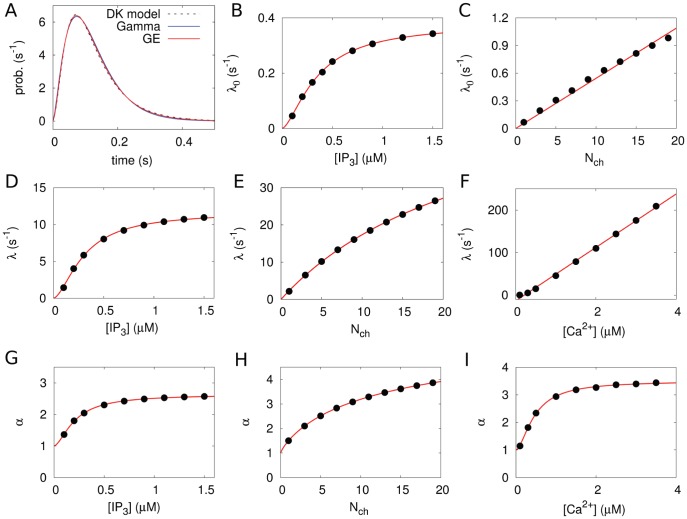
Parameter dependencies of the opening probabilities 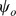. (A) Comparison of fits of Gamma and GE distributions to the De Young-Keizer model (see Ref. [34] and Table S1) at 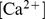 = 0.6 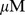. (B–I) Circles are fits of results from the De Young-Keizer model to GE distributions (Eq. 3) with parameters 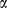 and 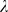, as in A. Lines are (B,D,E) nonlinear fits to Eq. 4, (C,F) linear fits, (G–I) nonlinear fits to Eq. 5. (B–C) show the dependencies for the puff-rate, i.e. at base-level 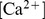 = 0.1 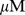 (see Text S1). (D–H) show parameter dependencies in the regime of a puff, i.e. with one nearby cluster already open, yielding 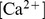 = 0.6 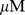 (see Text S1). Other parameter values (if not varied on the x-axis) are 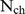 = 5, 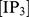 = 1 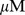.

All parameters that modify properties of 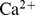 diffusion in the cell and describe the spatial configuration of clusters affect the coupling [14]. Therefore, we seek for a valid analytical approximation of 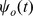 and its dependence on 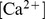, 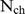 and 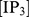, which was not available in our previous article [17]. Recent data from TIRF microscopy revealed key properties of 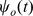 [27], [40], but are not sufficient for a closed mathematical model, because the dependencies of the 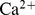 and 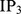 concentration have not yet been established. A good approximation was achieved by using two-parametric probability distributions like the waiting time density of the inhomogeneous Poisson process or the 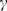-distribution, indicating recovery times between successive opening events [27]. However, in some cases, simple exponential distributions without any recovery process described the data equally well [27], [40]. This coincides with our observation that single channel models generate approximately exponential distributions at low (basal) local 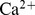 concentration [17]. This is probably because intermediate inhibitory states are rarely reached at low 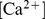 in these models [14]. Based on these findings, we assume for the purposes of this study that the transition from 0 to 1 open clusters (puff probability) is governed by a pure exponential distribution with average interpuff interval as recorded for SH-SY5Y cells for typical values of cellular parameters. For subsequent cluster openings (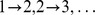 open clusters), we use GE distributions (see Eq. 3), which fit the data equally well as 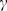-distributions (Fig. 2A) but are more convenient for operations in Laplace space. That means, for the puff probability distribution, we set 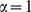 and identify 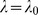 with the puff rate.

As we have determined the functional form of the 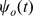, it remains to establish the dependencies of the distribution parameters 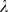 and 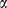 on the cellular parameters 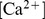, 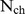 and 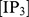. Unfortunately, for such dependencies no directly usable experimental data are available. However, earlier work has shown that 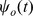 can be computed from single channel models by solution of a Markovian master equation [41]. The single channel models were partly based on data from *in vitro* studies, and the new *in vivo* experimental data [27] confirmed the type of distributions resulting from those models. In particular, we adopt an implementation of the De Young Keizer model [34], [41], one of the most accepted single channel models in the field (see Table S1 and Text S1 for details). To obtain an analytical description of the parameters 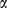 and 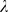, we performed simulations of the De Young Keizer model with varying cellular parameters and inspected functional forms matching the simulations. The base-level 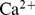 concentration, an important (and experimentally undetermined) parameter needed for the simulations, was chosen by comparison with the measured puff rate [27].

We found (Fig. 2B–C) that the parameter dependencies of the puff rate 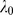 can be approximated by a Hill function.
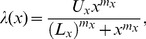
(4)where 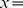
[IP_3_] or 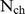. Note that 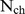 is a discrete variable, and Eq. 4 should only be evaluated at integer values in this case. This is also true for the dependencies of the parameter 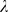 of the GE distribution on 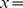
Ca^2+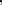
^[IP_3_]
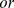
N_ch_ (Fig. 2D–F). Dependencies of 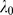 on 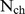 and of 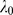 on 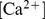 are close to the linear regime (Fig. 2C,F). The 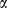-dependence of all parameters (Fig. 2G–I) can be fit by another Hill equation,
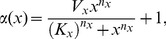
(5)where the ‘+1’ in Eq. 5 reflects the fact that GE-distributions have a different shape at 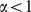. The fits of analytical functions to computed opening probabilities 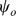 at varying cellular parameters provide all the information needed to perform calculations of the hierarchic stochastic model (see Text S1). Fit parameters for the examples shown in Fig. 2 are given in Table S2.

The analytical parameter dependencies of waiting times presented here are a huge advantage over the approach followed in Ref. [17], where the single channel models were evaluated de novo for each of model simulation at a certain set of cellular parameters. In particular, Eqs. 5–4 allow for integration of the hierarchic stochastic model into models of signal transduction networks in future research.

### Analysis of a Minimal Mechanistic 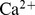 Model

Solution of the hierarchic stochastic model by the Laplace transform is most convenient in the case of a linear chain (see Section Materials and Methods). As applied to stochastic 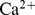 dynamics, cluster arrangements with equal distances between all clusters and identical clusters lead to linear chain models. That can be achieved only up to 4 clusters in a tetrahedral arrangement, but this number of clusters leads to physiologically meaningful results concerning spike statistics already [17], in particular in conjunction with the generic model introduced below. Further details needed to derive the 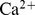 model are given in Text S1. By using the analytic approximations, Eqs. 3–4, we can relate cellular parameters like the cytosolic 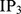 concentration to the parameters of the opening and closing waiting time densities.

**Figure 3:**
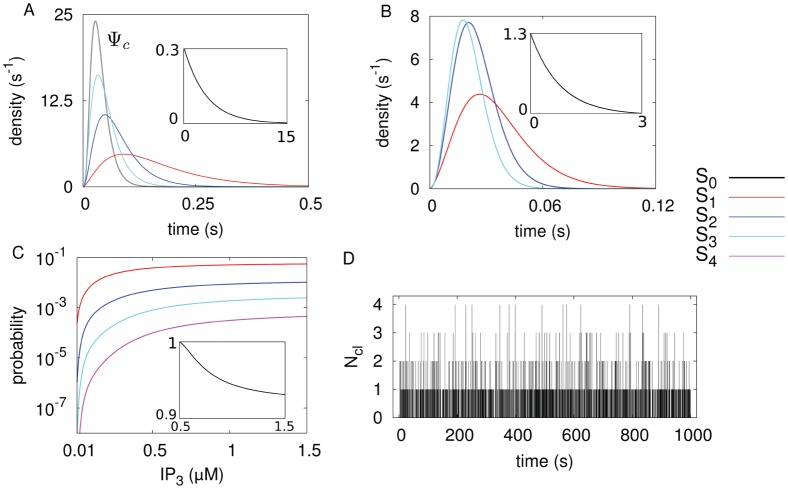
Minimal hierarchic stochastic model of 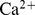 dynamics. The colours in (A,B,C) depict the system state in terms of the number of open clusters (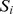, i = 0,1,2,3,4). (A) Individual opening time (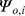) and closing time (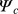) probability densities. (B) Conditioned waiting time densities for the consecutive opening transitions, constructed out of the individual closing (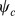) and opening (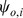) densities for the tetrahedron model. Note the time-scale difference between the opening of the first cluster (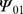) and the consecutive openings, this effectively covers the CICR mechanism. (C) Stationary probabilities for the possible states of the system as function of the cytosolic 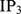 concentration, as computed from Eq. 14. (D) Stochastic simulations of spike trains for 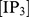 = 1 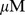. Note the frequent puff events with less than four clusters open and the rather isolated global spike events. The number of channels per cluster is 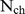 = 5. All calculations are based on the analytic approximations of parameter dependencies shown in Fig. 2.

Figure 3 shows a typical model calculation with realistic parameters and spiking statistics. The opening probabilities (Fig. 3A–B) are split into two temporal regimes for opening of the first cluster with the constant rate 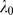 (puff probability, inset) and successive cluster openings. If the first cluster has opened, the probability for the opening of successive clusters is greatly enhanced. This reflects CICR in our stochastic model. The stationary occupancy probabilities (Fig. 3C) for the different states can be computed from Eq. 14. We performed delayed stochastic simulations (Fig. 3D) by methods similar to the Gillespie algorithm [13], [17] to provide a picture of the actual dynamics.

**Figure 4:**
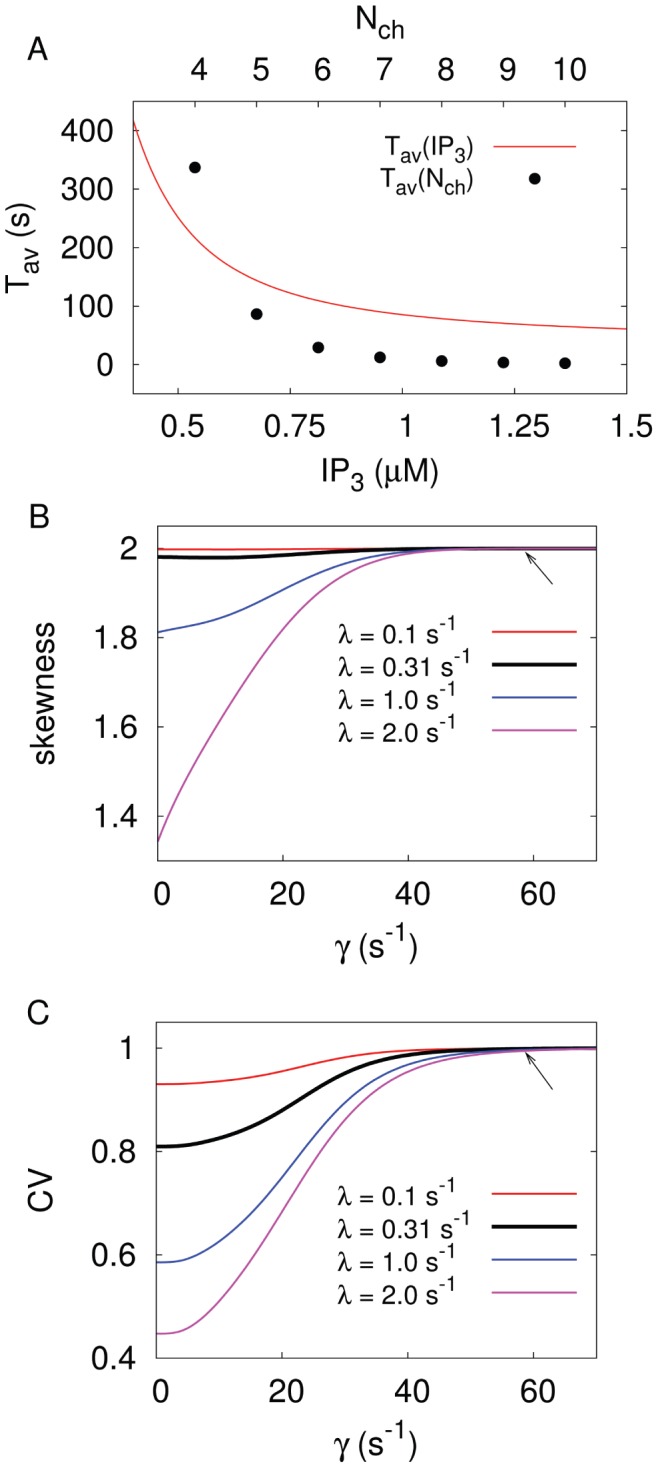
Statistical analysis of 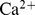 interspike intervals (ISI). (A) The average ISI 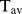 decreases with the 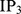 concentration (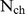 = 5) and with 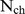 (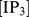 = 1 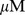 ). The other parameter is kept at 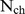 = 5 and 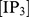 = 1 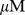, respectively. (B) Skewness and (C) coefficient of variation (CV = standard deviation/mean) increase with the channel closing rate 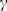, which controls the time scale separation between 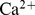 puffs (single cluster opening) and 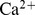 spikes (all clusters open). The solid lines are calculated for a realistic puff rate 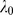. The dotted lines indicate that the time-scale separation becomes weaker with growing puff rate (see text). The parameter values used for the stochastic 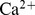 model (Fig. 3) are indicated by the arrows.

We define a spike in the tetrahedron model by opening of all four clusters. The average ISI 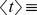
T_av
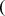
_[IP_3_]
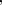
N_ch
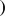
_ can then be calculated analytically by Eq. 15. Figure 4A shows that 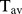 changes by about one order of magnitude with varying 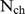 and 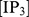, respectively. If we suppose a realistic coverage of the cellular parameter range, a strong cell to cell variability in terms of 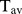 should be expected in experiments, and is indeed reported [6], [26]. Even though we can not calculate the ISI density directly, apart from very simple cases (see Text S1) where the inverse Laplace transform is feasible, we can characterise it with higher moments, which are directly available from our theory (see Methods, Eq. 15). For standard cellular parameters as used above, we obtain a skewness of 2 (Fig. 4B) and an excess kurtosis of 6. These are exactly the values fulfilled by an exponential distribution, and indeed 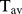 (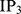, 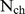 ) = 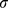 (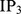, 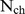 ) also holds for our results (Fig. 4C). Thus, we conclude that the tetrahedron model yields an exponential ISI density in the realistic parameter regime.

The surprisingly simple result of an exponential ISI density for a large range of the biological parameters is due to the strong time-scale separation between the relatively slow puff dynamics and the faster excitation process up to the spiking state (see Fig. 3A). If a puff occurs the system decides rather quickly, in comparison to the interpuff intervals, whether it generates a global spike or relaxes back to the ground state with all clusters closed. Hence, the system spends most of its time in the ground state, waiting for the next puff to occur, and the constant puff probability dominates the ISI density. The ISI density is different from an exponential distribution for smaller time scale separation, i.e., smaller values of 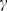 (Fig. 4B–C). Remarkably, we find parameter regions where the coefficient of variation (CV = standard deviation/average) drops significantly whereas the input distribution (puff-distribution) is purely exponential (CV = 1). This indicates array enhanced coherence resonance [42], [43], a phenomenon that has been intensively studied in physics and is a characteristic of coupled noisy excitable systems. See Text S1 for a simple example where array enhanced coherence resonance can be demonstrated analytically.

The exponential dependence on time of the ISI density entailing 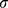 = 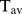 is quite robust with respect to even strong variations of the puff rate with realistic channel closing rates (arrows in Fig. 4B–C). This is in agreement with our earlier result [17] that a global feedback is necessary to reach the regime 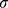
<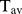, which is typically observed in experiments [6]. Unfortunately, global feedback does not allow for an exact analytical solution for the moments of the FPT density. However, based on the robust time-scale separation observed in the mechanistic model (Fig. 4), we found a valid approximation of the spike process incorporating a global feedback and the local dynamics, which we call generic model.

### A Generic Model Based on Local Puff Dynamics

We use the simple model for the global spike process introduced in Ref. [6] to introduce a global feedback into the hierarchic stochastic process. The global feedback is included as a slow recovery of the spike rate after a spike occurred, which leads to an inhomogeneous Poisson process with time dependent spike rate.
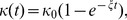
(6)with the Poisson spike rate 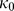 and the recovery rate 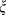. With this formulation the probability for a spike at 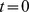 equals zero. This represents an inhibitory or negative global feedback, at high 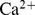 concentrations, which naturally occurs after a global 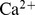 spike.

**Figure 5:**
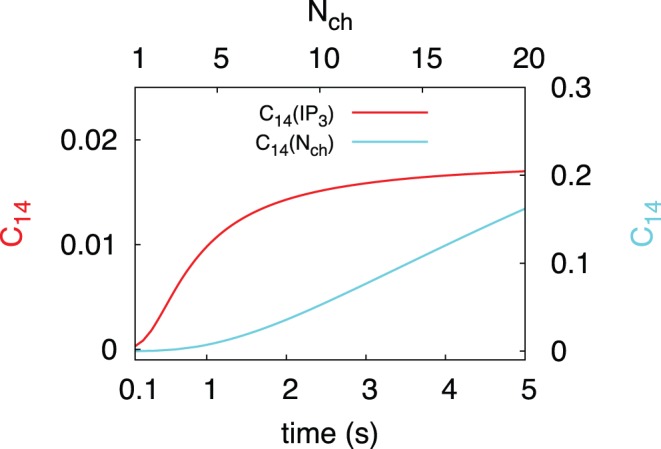
Parameter dependencies for the splitting probability 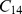 to reach the spiking state 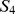 out of a single puff state 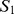 for the tetrahedron model. Note the different axis scaling for the cytosolic 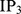 concentration and the mean number of 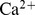 channels per cluster participating in an opening event (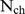 ) respectively. The splitting probability saturates for both parameters, however for the 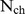 dependency only for a unrealistic high number of 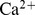 channels (N_ch
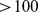
_).

The probability for a spike is the probability for a puff times the probability that the puff opens the other clusters. The latter is in mathematical terms a splitting probability that a single puff triggers a consecutively opening of all clusters. For the tetrahedron model this means to go from one open cluster to four open clusters before reaching the ground state with all clusters closed again. It is analytically given by [17].
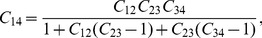
(7)with the one-step splitting probabilities 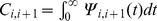. Figure 5 shows the dependence of 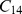 on cellular parameters. As in Ref. [17], we formulate puff generation as inhomogeneous Poisson process with time-dependent rate 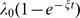, where 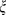 is the recovery rate. This effectively inhibits the occurrence of a local puff event right after a spike and hence incorporates global negative feedback. The puff rate asymptotically approaches the stationary value 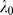 as time increases. We now resample the puff process into the global spike process with probability 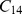 and its complementary process with probability 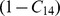. The complementary process is the Poisson process describing the failed puffs, the ones that did not lead to a global 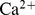 spike.

In general, a cellular spike will require N open clusters, the splitting probability should therefore be denoted by 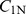. Clusters are heterogeneous with respect to the number of channels per cluster as well as to their distance to neighbouring clusters. Hence, the puff probability as well as the splitting probability are cluster specific. We introduce the individual puff rate 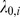 and splitting probability 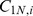 for every cluster 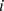. Some failed puffs occurs between two spikes. The probability for the interspike interval 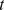 is then given by the sum over the probabilities for all possible numbers of puffs in between two spikes from 0 to 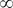. This finally leads to the expressions constituting the inhomogeneous Poisson splitting (see also Text S1).
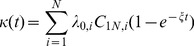
(8)

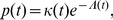
(9)where 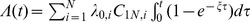 is the intensity and 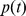 is the density function of the probability that a global 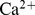 spike occurs at time 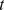 after the previous spike.

Comparison with Eq. 6 shows that we found an expression of the Poisson spike rate 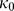 in terms of cluster properties,
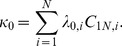
(10)


Thus, 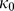 can be taken as total spike rate, summing up the individual contributions of clusters 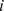 to the spike rate. That is, the individual puff rates 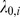 of cluster 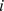 are rescaled with the coupling strength 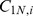. The average interspike interval 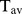 and the standard deviation 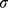 can then be calculated analytically with the results given in Ref. [6].

**Figure 6:**
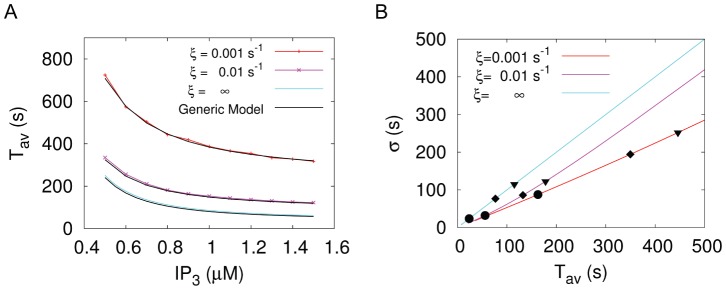
The generic model is a valid approximation of the hierarchic stochastic model and provides 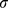
–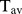 relations close to experimental data. (A) Comparison of numeric results for the tetrahedron model with the results for 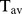 of the generic model with different feedback strengths represented by 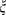 (see also S1). For 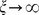, the model approaches the analytically treatable case, where the slope of the relation equals one. (B) 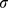
–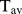 relation of the generic model. To demonstrate the impact of the coupling strength 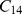, we added the individual values of 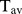 for three different values of 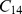 (0.05, 0.01, 0.008) to each line. Modification of the coupling strength merely leads to a shift of the cell along the 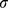
–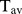 relation and does not affect the slope of the relation.

To confirm the validity of the generic model in the presence of global feedback, we performed stochastic simulations of the hierarchic stochastic model as described in Ref. [17] and compared them with the analytic results of the generic model. For the homogeneous tetrahedron cluster arrangement, Eq. 10 gives 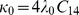. This new generic model approximates the average ISI very well (Fig. 6A). The experimental moment relations are in very good agreement with the moment relations obtained for 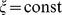 (Fig. 6B). For a single cell the specific value of 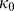 positions the cell on that moment relation as indicated by the symbols in Fig. 6. Note that very small slopes of the moment relation below 0.3, which have been reported for HEK293 cells [6], can be generated by adding cooperativity to the generic model [18]. The saturation for both 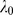 and 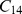 with respect to the 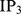 concentration (see Figs. 2 and 5) gives rise to an intrinsic minimal ISI in the model (Fig. 6A–B). The relation of this minimal ISI to the physiological minimal ISI reported from single cell measurements [6] should be elucidated by further research.

By applying Poisson splitting (see Text S1 for details), we implicitly neglect the possible dynamics between the puff and the spike events. Most notably we completely miss the duration of the failed puffs. The number of those failed puffs is given on average by 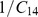, so we expect the error we make by using the splitting scales exactly with 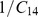. By comparing our exact analytic results for the tetrahedron model with our generic model without recovery we show that this is indeed the case (Fig. S1). Moreover we have computed the relative error and state that it is bounded and smaller than 10% (see Text S1). The great advantage of the generic model is its capability of providing a closed expression for the ISI distribution reproducing the moment relation between the standard deviation 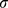 and the average 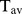 of ISIs found experimentally, incorporating the local puff dynamics. The key parameter for the local dynamics is the coupling strength 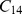 obtained by hierarchic stochastic modelling.

## Discussion

We present an extension to a description of complex stochastic networks in terms of observable states and non-exponential transition time distributions. The approach can be used for very efficient stochastic simulations and in many cases analytical solutions can be obtained. We apply hierarchic stochastic modelling to spatially resolved stochastic 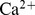 dynamics. Apart from using a new solution method, we made further progress in the 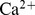 model published in [17] by developing analytic approximations of model dependencies on cellular parameters and taking into account recent experimental data for model calibration. This permitted us to rationalise time scale separation between puffs and waves in a physiological setting, and subsequently to find a derivation of a generic description of the stochastic spike generating process.

The theory presented here is able to calculate the statistical properties of cellular 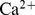 spiking from cluster properties and spatial coupling. We have derived the moments of the ISI distribution by Laplace transform of the non-Markovian master equation, and we approximated the complete ISI distribution with the generic model. This simplistic description was postulated earlier in the Falcke group [6] but only now we can give a mechanistic explanations for its validity. The generic model is only applicable for a large time scale separation between the interpuff intervals and the ISIs. However, this separation was shown experimentally for many cell types [27], [44]. As is often the case with approximate solutions, the generic model allows for deeper insight than the direct solution of the integro-differential equations. The theory explains the role of spatial arrangement of clusters and strength of spatial coupling in the spike probability in a simple and intuitive way. We introduced a phenomenological factor 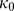 which rescales the local puff dynamics to the global spike dynamics. This factor also explains the observed large cell-to-cell variability as differences in cluster properties and cluster arrangement. While we started with the assumption that the puff probability slowly recovers from negative feedback, Eqs. 8 also allows for a time dependency of the splitting probability 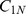. Hence, more complicated recovery processes arising from a variety of feedbacks could replace the simple relaxation chosen here. The model exhibits almost linear moment relations between standard deviation and average in agreement with experimental results. Changes in 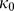 of an individual cell by experimental manipulations or between cells due to cell-to-cell variability do not alter the slope of the moment relation. That explains its robustness against changes of local properties and coupling strength, i.e. cell-to-cell variability within a given cell type, which has been observed [17], [32]. The rate of recovery from global feedback strength 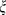 is cell type specific, which explains the cell type specificity of the moment relation.

Our theory is based on recent experimental data from live cell imaging for opening and closing transition times of 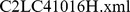 channel clusters. A description of cellular 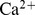 dynamics further requires information about the coupling of individual 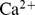 channel clusters and the dependence on cellular parameters like strength of the upstream signal (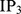 concentration) and cluster size. These functional relations could, up to now, only be obtained from single channel models, which are well established and have successfully been used in earlier modelling studies [14], [17], [32]. Based on this, we suggest analytical approximations for model dependencies on cellular parameters. This approach can be used for integration of the model into mathematical descriptions of larger signalling cascades.

We calculated the splitting probability 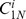 for 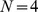, where 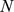 is the number of open clusters forming a cellular release spike. Typically, a cellular spike will consist of more than 4 open clusters. However, the calculation provides a good description of cellular spiking, if a cell has a preferred nucleation area initiating most of the cellular spikes. The stronger the release in such an area is, the more likely is the nucleation of a wave due to 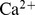 diffusion and CICR. At a sufficient value, this probability will be very close to one. The tetrahedron model is a good description of cellular spiking with nucleation areas, if that value is reached by 4 open clusters. Preferred nucleation areas have indeed been observed, e.g. in hepatocytes [45]. In the spirit of hierarchical modelling, the results with one nucleation area can then easily be generalised to several such areas.

We find that the minimal tetrahedral system considered here stays in the regime of a Poisson process for 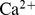 spikes, characterized by a coefficient of variation equal to one, even for relatively large deviations from the physiological parameters. Only a drastic reduction of the time scale separation (or increase of the coupling strength between clusters), mediated by increased puff rate 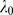 and reduction of the channel closing rate 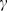, can reduce the noise level by means of array enhanced coherence resonance [42], [43]. Therefore, we suppose that the lower noise levels observed in many cell types [6] indeed stems from feedback processes, as suggested by earlier results [17]. However, the experimental proof for the importance of such feedback processes for 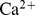 spike statistics is still lacking. Given an inhomogeneous Poisson process for the formation of a puff, the subsequent nucleation process (one to many open clusters) is very fast compared to a typical ISI. This time scale separation is the key assumption behind the generic model. By incorporation of a negative feedback it leads to ISI distributions close to experimental data.

Hierarchic stochastic modelling uses non-exponential transition times to reflect microscopic state changes occurring during sojourns in observable states. Remarkably, such non-exponential transition times have already been measured or computed for other cellular systems like gene expression [37], [38], autocatalytic reactions [46], [47] or signal transduction cascades [48], [49]. Using non-exponentially distributed transition times instead of kinetic constants can also be interpreted as dynamic modelling with stochastic delay – the delay arises from the underlying microscopic dynamics (see Fig. 1). Biological systems with stochastic delay were previously investigated [50]–[53], but these studies lack the possibility to implement molecular interactions on the lumped state level, and have not been generalised to hierarchic networks. Hierarchic stochastic modelling can also be related to published methods which exploit the hierarchic structure of networks [54]–[56]. Similar to our approach, such methods reduce the state space based on emergent behaviour of biomolecules arising by the dynamic hierarchy from molecular interactions to cellular properties [19], [57], [58]. However, to our knowledge, these studies have been limited to a reduction of the state space or inference of system parameters in deterministic systems so far. Since complex stochastic networks are frequently observed in cell biology [1], [2], we expect that our approach has a wide range of potential applications. On the one hand, pure Markov modelling means that one has to take into account all microscopic variables to make an exact description of the system. This is impossible for most, if not all, processes analysed in cell biology due to the large numbers of system states and because microscopic state transitions can rarely be measured *in vivo*. On the other hand, the assumption of Gaussian white noise, which is often used in approximation techniques, is questionable for many biological systems because the number of independent identical processes is too small for application of the law of large numbers. Therefore, hierarchic stochastic modelling brings theoretical concepts closer to experimental data.

The application to intracellular 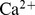 dynamics presented here makes an important step forward in the development of a mechanistic but comprehensive mathematical description of the process. We are now in a position to characterise the stochastic process in the physiologic parameter regime. A key finding is that the time scale separation between single cluster puffs and subsequent openings is very strong. And that despite the non-Markovian nucleation process, a coefficient of variation smaller than one can not be realised without a negative feedback. These results pave the road to the generic description in the form of a time-dependent Poisson process, which can be sampled by standard methods. Our derivation shows how the phenomenological parameters of the generic model depend on cellular parameters. The generic model can therefore be used to generate 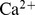 spikes with correct statistics in models of larger signalling cascades in future research.

## Materials and Methods

### The Conditioned Waiting Times 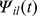


In the mechanistic stochastic 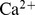 model recently published by two of us [17], we not only lumped the microscopic states of individual 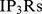 but the whole ensemble of 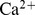 channels forming a cluster. Therefore, our effective reduced state space just contains the time dependent cluster configuration, i.e. the number of open and closed 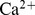 clusters at a specific time point. In the theoretical framework of semi-Markov processes (see Text S1 for details) transitions within that state space can be described by the probability given by 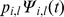. The first factor describes the transition probability of an ordinary discrete time Markov chain for the transition 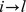, the latter gives the possibly non-exponential waiting time distribution. The 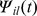 are not true probability densities for more than two possible state transitions, as they are not normalised individually, but rather by the condition 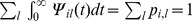. Therefore the term conditioned waiting times (or conditioned dwell times) is appropriate. The waiting times can be calculated from a mathematical model or measured directly (see Section Results). Exact definitions of the 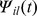 and for the considered semi-Markovian stochastic process are given in Text S1. Apart from the analytical approach given below, the 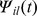 can also be used very efficiently in stochastic simulations [17].

### Solution of the Non-Markovian Master Equation

State-transition with non-exponential waiting times are not covered by Eq. 1, but rather by a generalised (non-Markovian) master equation [17], [59].
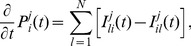
(11)with the probability fluxes given by [59]



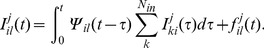
(12)Note that the probability fluxes 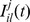 can no longer be expressed in terms of the 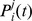 like in Eq. 1, but are solutions of a Voltera integral equation determined by the conditioned waiting times 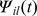 and appropriate initial functions 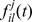. The initial functions are related to the initial probabilities 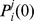 in Eq. 11, e.g. 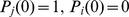 sets 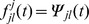 and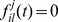 for all 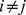 and all 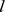.

The form of Eq. 11 suggests a solution based on the Laplace transform 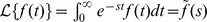 (see also [60]), because the convolution theorem implies.
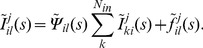
(13)


This constitutes a linear system of equations for the probability fluxes in Laplace space. We obtain the stationary occupancy probabilities as.
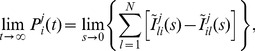
(14)where the limits in Eq. 14 are equal due to the final value theorem of the Laplace transform.

Certain dynamical system properties can be characterised by statistical moments of the density function of the first passage time (FPT) 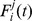 giving the probability for the first visit of state 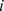 when starting at state 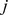. The moments of 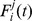 obey [11]:
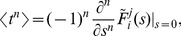
(15)where the Laplace transformed FPT density can be found by the formula 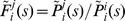 [11]. By using the property 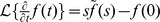, the next step is to apply the Laplace transform to Eq. 11. This yields
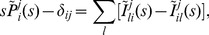
(16)stating that 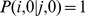 for 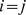 and zero otherwise. From this, we find



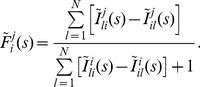
(17)This expression is particularly simple in the case of a linear chain with 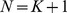 distinct states 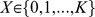, where we have to compute the 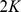 probability fluxes 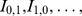 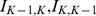. Suppose the system is in state 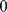 at time 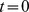 and we want to calculate the moments of the FPT distribution to reach the highest state 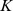 using Eq. 15 derived above. In the case of a linear chain, we arrive at
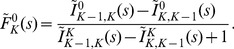
(18)


The conditioning on either state 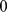 or state 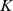 at 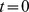 leads effectively to two distinct non-homogeneous linear systems of equations for the fluxes. They differ only with respect to the initial functions, namely 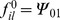 and 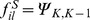. Therefore, in the case of 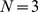 we obtain Eq. 2 (see Text S1 for details).

### Model Calculations

Details of the mechanistic 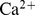 model are given in Text S1. We used Wolfram’s Mathematica 8 for linear algebra and a home-made simulation tool [17] for stochastic simulations.

## Supporting Information

Figure S1
**Comparison of the generic model with the exact analytic results provided by the hierarchic stochastic model.** Shown are the results for the realistic channel closing rate 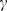 (black lines) and two examples with lower time scale separation. (A) The approximation error grows linear with the average number of failed puffs, given by 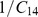. (B) The relative approximation error is smaller than 10% for the realistic closing rate.(PDF)

Table S1
**Parameter values for the De Young-Keizer model.** The De Young-Keizer model with the parameters in this table is used to compute the opening transition times 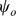.(PDF)

Table S2
**Analytical approximation of the GE distribution parameters.** The values in this table result from nonlinear fitting to Eqs. 4–5 (see Fig. 2) and are used for the dependencies of the GE distribution parameters 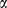 and 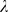 on cellular parameters 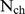, 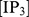 and 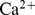 (see Text S1 for details). In each row, only one parameter is varied, and other parameters are kept constant at 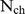 = 5, 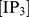 = 1 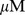, and 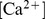 given by Eq. 41 in Text S1. Every system state is described by the number of open clusters (o. cl.) and has its own set of parameters.(PDF)

Text S1 (PDF)
