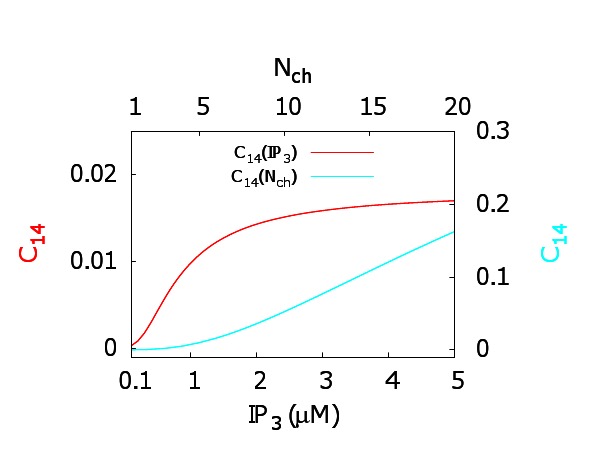# Correction: Hierarchic Stochastic Modelling Applied to Intracellular Ca^2+^ Signals

**DOI:** 10.1371/annotation/55c7cbd5-433c-46e6-a44e-c73f66eae122

**Published:** 2013-06-06

**Authors:** Gregor Moenke, Martin Falcke, Kevin Thurley

The correct name of the third author is: Kevin Thurley

The correct version of Figure 5 available here: 

**Figure:**