# Supplementary figures and images for: Hierarchic Stochastic Modelling Applied to Intracellular Ca2+ Signals
Source: PLoS One. 2012 Dec 27;7(12):e51178. doi: 10.1371/journal.pone.0051178 (PMC3531454; doi:10.1371/journal.pone.0051178)

A

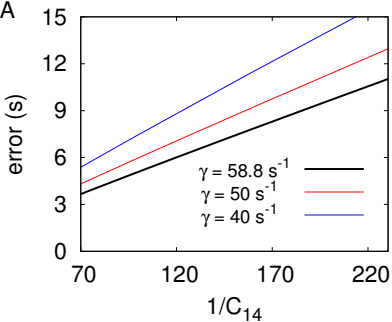

B

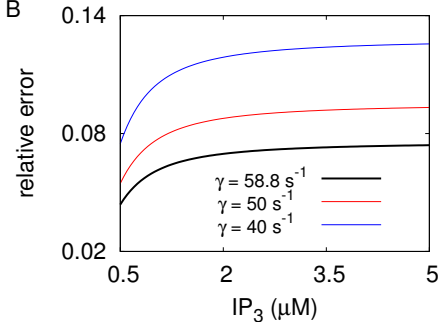

Supplement: Figure S1 — Comparison of the generic model with the exact analytic results provided by the hierarchic stochastic model. Shown are the results for the realistic channel closing rate (black lines) and two examples with lower time scale separation. (A) The approximation error grows linear with the average number of failed puffs, given by . (B) The relative approximation error is smaller than 10% for the realistic closing rate. (PDF) [file pone.0051178.s001.pdf]
