# Supplementary material for: Hierarchic Stochastic Modelling Applied to Intracellular Ca2+ Signals
Source: PLoS One. 2012 Dec 27;7(12):e51178. doi: 10.1371/journal.pone.0051178 (PMC3531454; doi:10.1371/journal.pone.0051178)
Supplement: Table S1 — Parameter values for the De Young-Keizer model. The De Young-Keizer model with the parameters in this table is used to compute the opening transition times . (PDF) [file pone.0051178.s002.pdf]

Table S1: Parameter values for the De Young-Keizer model used to compute the opening transition times ψ_o.

**Parameter**	**Value**	**Unit**
a1	20	$(\mu M\ s)^{-1}$
a2	0.001	$(\mu M\ s)^{-1}$
a3	20	$(\mu M\ s)^{-1}$
a4	0.025	$(\mu M\ s)^{-1}$
a5	10	$(\mu M\ s)^{-1}$
d1	2.6	μM
d2	0.03077	μM
d3	2.6	μM
d4	0.1	μM
d5	12.25	μM
