# Supplementary material for: Hierarchic Stochastic Modelling Applied to Intracellular Ca2+ Signals
Source: PLoS One. 2012 Dec 27;7(12):e51178. doi: 10.1371/journal.pone.0051178 (PMC3531454; doi:10.1371/journal.pone.0051178)
Supplement: Table S2 — Analytical approximation of the GE distribution parameters. The values in this table result from nonlinear fitting to Eqs. 4–5 (see Fig. 2) and are used for the dependencies of the GE distribution parameters and on cellular parameters , and (see Text S1 for details). In each row, only one parameter is varied, and other parameters are kept constant at = 5, = 1 , and given by Eq. 41 in Text S1. Every system state is described by the number of open clusters (o. cl.) and has its own set of parameters. (PDF) [file pone.0051178.s003.pdf]

Table S2: Analytical approximation of the GE distribution parameters.

	$V_{Ca^{2+}}$	$K_{Ca^{2+}}$ (μM)	$n_{Ca^{2+}}$	$U_{Ca^{2+}}$ ($\mu M^{-1}s^{-1}$)	$L_{Ca^{2+}}$ (μM)	$m_{Ca^{2+}}$
	2.503	0.467	1.662	625.400	5.569	1.500
o. cl.	V_{IP_3}	K_{IP_3} (μM)	n_{IP_3}	U_{IP_3} ($\mu M^{-1}s^{-1}$)	L_{IP_3} (μM)	m_{IP_3}
0	0.384	0.358	1.550	—	—	—
1	1.636	0.211	1.609	11.983	0.319	1.542
2	2.332	0.196	1.653	27.617	0.292	1.496
3	2.650	0.190	1.665	43.589	0.282	1.466
o. cl.	$V_{N_{ch}}$	$K_{N_{ch}}$	$n_{N_{ch}}$	$U_{N_{ch}}$ (s^{-1})	$L_{N_{ch}}$	$m_{N_{ch}}$
0	0.056	—	—	—	—	—
1	4.952	13.130	0.850	66.974	27.877	0.986
2	5.229	7.555	0.820	130.760	27.691	0.882
3	5.319	5.847	0.803	198.814	28.555	0.837
