# Supplementary material for: Hierarchic Stochastic Modelling Applied to Intracellular Ca2+ Signals
Source: PLoS One. 2012 Dec 27;7(12):e51178. doi: 10.1371/journal.pone.0051178 (PMC3531454; doi:10.1371/journal.pone.0051178)
Supplement: Text S1 — (PDF) [file pone.0051178.s004.pdf]

Supporting Text S1 for Manuscript: Hierarchic stochastic modelling of observable cellular dynamics

Gregor Moenke, Martin Falcke, Kevin Thurley

This document contains the following information:

1. Detailed mathematical definitions for the semi-Markov process and the conditioned waiting times used for hierarchic stochastic modelling.
2. Explicit derivation of the First Passage Time for a discrete state semi-Markov system by using Laplace transformed probability fluxes.
3. Exemplary demonstration of the derived First Passage Time formalism for a three state Markov model.
4. Laplace transformation of the generalised exponential distribution
5. Details for the minimal mechanistic Ca^{2+} model, including derivations of the waiting time densities and their dependencies on cellular parameters.
6. Detailed derivation of the generic Ca^{2+} model.
7. Comparison of the generic Ca^{2+} model with the exact analytic results provided by the hierarchic stochastic Ca^{2+} model.

1 Semi-Markov processes

We define an appropriate stochastic process $X_n(t)$ we can use as a model for the intracellular calcium dynamics. We start with a finite sample space $\Omega = \{0, 1, ..., S\}$ consisting of all possibly macroscopic states the process can visit. By $T_n \in \mathbb{R}^+$ we denote the subsequent transition epochs between these states with $T_0 < T_1 < ... < T_n$. We can now formulate transition probabilities according to

$$Q_{i,j}(t) = \mathrm{P}\{X_{n+1} = j, T_{n+1} - T_n \leq t | X_n = i\}. \qquad (1)$$

The process is temporally homogeneous by noting the independence of $Q_{i,j}(t)$ from n, and we also assume $Q_{i,j}(0) = 0$.

A process which is governed by Eq. 1 is called a semi-Markov process [1–3], with $Q = \{Q_{i,j}(t); i, j \in \Omega, t \in \mathbb{R}^+\}$ forming a so called semi-Markovian kernel. We will briefly summarise the reasoning behind that terminology. By defining

$$p_{i,j} = \lim_{t\to\infty} Q_{i,j}(t) \qquad (2)$$

one gets the transition probabilities for the embedded Markov chain with normalisation condition $\sum_j p_{i,j} = 1$. Next we define the following probability distributions:

$$G_{i,j}(t) = \frac{Q_{i,j}(t)}{p_{i,j}} = \mathrm{P}\{T_{n+1} - T_n \leq t | X_n = i, X_{n+1} = j\}. \qquad (3)$$

The successive visits of the process X_n form a Markov chain with transition probabilities $p_{i,j}$, whereas the length of the sojourn time intervals $[T_n, T_{n+1})$ are given by the distribution functions $G_{i,j}(t)$. If these distributions can be written as $G_{i,j}(t) = 1 - \exp(-\sum_j q_{i,j}t) \equiv G_i(t)$, than the process is a pure Markov process with rates $q_{i,j}$, the sojourn times are exponentially distributed and independent of the next state. In that case the process is memoryless, which means that

$$\mathrm{P}\{T_{n+1} - T_n > s + t | T_{n+1} > t\} = \mathrm{P}\{T_{n+1} - T_n > s\}, \qquad (4)$$

for $s, t > 0$ only holds for exponential distributions.

In conclusion, a semi-Markov process (X_n, T_n) still fulfils the Markov property with respect to the subsequent state transitions, but allows arbitrary (with respect to $\sum_j \lim_{t\to\infty} Q_{i,j}(t) = 1$) sojourn time distributions and hence looses its memorylessness with respect to the transition times $T_{n+1} - T_n$. It is therefore the ideal frame work to apply the desired non-exponential waiting times often

found experimentally, now exactly defined by:

$$\frac{d}{dt}Q_{i,j}(t)dt = \Psi_{ij}dt = \mathrm{P}\{t < T_{n+1} - T_n < t + dt | X_{n+1} = j, X_n = i\}. \tag{5}$$

Therefore, as introduced in the main text, the term conditioned waiting time seems appropriate. By noting that the transition probabilities of the embedded Markov chain are given by $p_{i,j} = \int_0^\infty \Psi_{i,j}(t)dt$, the semi-Markov process is completely defined by a set of conditioned waiting time densities. Also note that by definition of the transition probabilities in Eq. 1, the time variable t does not correspond to a system time, but describes the wait after the last transition T_n.

2 Detailed derivation of the first passage time density

We want to outline the computational strategy to compute mean first passage times (FPT) for arbitrary discrete state semi-Markovian systems. The particular simple case of a linear chain is used for the stochastic Ca^{2+} model, and we will show its solution as a special case at the end of this section.

In the main text we introduced the Laplace transformed non-Markovian master equation:

$$s\tilde{P}_{i,j}(s) - \delta_{ij} = \sum_l \tilde{I}^j_{li}(s) - \sum_l \tilde{I}^j_{il}(s), \tag{6}$$

containing the Laplace transformed probability fluxes $\tilde{I}^j_{il}$. The FPT is given by (see also main text)

$$\tilde{F}_{i,j}(s) = \frac{\sum_l \tilde{I}^j_{li}(s) - \sum_l \tilde{I}^j_{il}(s)}{\sum_l \tilde{I}^i_{li}(s) - \sum_l \tilde{I}^i_{il}(s) + 1}, \tag{7}$$

with the moments:

$$\langle t^n \rangle = (-1)^n \frac{\partial^n}{\partial s^n} \tilde{F}_{i,j}(s)|_{s=0}. \tag{8}$$

This equation yields for n=1 the mean FPT.

Next we calculate the Laplace transformed fluxes. We start with the formula for a single flux [4]:

$$I^j_{il}(t) = \int_0^t \Psi_{il}(t-\tau) \sum_k^{N_{in}} I^j_{ki}(\tau) d\tau + f^j_{il}(t), \tag{9}$$

which is a convolution of the conditioned waiting time to go to state l and all

incoming fluxes to the state i. The f_{il}^{j} are the initial functions, dealing with the initial state j of the whole system (see also main text). By writing all possible fluxes as a vector $\mathbf{I}^j(t)$ and the conditioned waiting time densities in an appropriate matrix $\mathbf{\Psi}(t)$, the system of integral equations can be written as

$$\mathbf{I}^j(t) = \int_0^t \mathbf{\Psi}(t-\tau)\mathbf{I}^j(\tau)d\tau + \mathbf{f}^j(t), \tag{10}$$

with $\mathbf{f}^j(t)$ as vector of the initial functions.

The transition network of the system is completely determined by the fluxes (see also [5]). A standard technique for solving such integral equations with a convolution kernel is by using the Laplace Transform $\mathcal{L}\{f(t)\} = \int_0^\infty e^{-st} f(t)dt = \tilde{f}(s)$, with the convolution theorem

$$\mathcal{L}\{f(t) * g(t)\} = \mathcal{L}\{f(t)\}\mathcal{L}\{g(t)\}. \tag{11}$$

Equation 10 reads in Laplace space:

$$\tilde{\mathbf{I}}^j = \tilde{\mathbf{\Psi}}\tilde{\mathbf{I}}^j + \tilde{\mathbf{f}}^j. \tag{12}$$

Basic algebra yields

$$\tilde{\mathbf{f}}^j = (\mathbb{1} - \tilde{\mathbf{\Psi}})\tilde{\mathbf{I}}^j. \tag{13}$$

This is an inhomogeneous linear system of equations for the Laplace transformed fluxes $\tilde{I}_{il}$ which can be solved exactly by standard algebraic methods. The solution yields all Laplace transformed fluxes as functions of the Laplace transformed waiting times. As can be seen in Eq. 7 for the FPT $\tilde{F}_{i,j}(s)$, there are two sets of fluxes needed, reflecting the initial states i or j respectively. Inserting these fluxes into Eq. 7 gives the desired Laplace transformed FPT density $\tilde{F}_{i,j}(s)$.

In case of a linear chain with $N = K+1$ states, we write for the flux vector $\mathbf{I}^j = (I_{01}^j, I_{10}^j, I_{12}^j, ..., I_{K-1,K}^j, I_{K,K-1}^j)$ and the matrix elements $\mathbf{\Psi}_{il}$ are determined by the set of equations given in Eq. 10 (see also section below for an example). The equation 7 for the FPT simplifies to:

$$\tilde{F}_{K,0} = \frac{\tilde{I}_{K-1,K}^0 - \tilde{I}_{K,K-1}^0}{\tilde{I}_{K-1,K}^K - \tilde{I}_{K,K-1}^K + 1}. \tag{14}$$

For our minimal stochastic Ca^{2+} model we have $N = 5$ and the FPT is given by:

$$\tilde{F}_{0,4} = \frac{\tilde{\Psi}_{01}\tilde{\Psi}_{12}\tilde{\Psi}_{23}\tilde{\Psi}_{34}}{1 - \tilde{\Psi}_{01}\tilde{\Psi}_{10} - \tilde{\Psi}_{12}\tilde{\Psi}_{21} - \tilde{\Psi}_{23}\tilde{\Psi}_{32} + \tilde{\Psi}_{01}\tilde{\Psi}_{10}\tilde{\Psi}_{23}\tilde{\Psi}_{32}}. \tag{15}$$

Note that we did not have to specify the explicit form of the conditioned waiting times for the entire calculation up to here. In order to calculate the mean FPT or higher moments of the FPT density Eq. 8 can be applied.

3 Minimal Markovian Example

The analytic approach presented in the last section for solving the first passage time problem shall be illustrated in a simple example. The system we want to consider is a three state continuous time Markov chain, with transitions

$$0 \underset{\gamma}{\overset{\lambda}{\rightleftharpoons}} 1 \underset{\gamma}{\overset{\alpha}{\rightleftharpoons}} 2\,. \tag{16}$$

We recall from the theory of continuous time Markov chains [6] that the sojourn time distributions are the following: $G_1(t) = 1 - e^{-\lambda t}, G_2(t) = 1 - e^{-(\alpha+\gamma)t}$ and $G_3(t) = 1 - e^{-\gamma t}$. By considering the transition probabilities of the embedded (discrete time) Markov chain, namely $p_{01} = 1$, $p_{10} = \frac{\gamma}{\alpha+\gamma}$, $p_{12} = \frac{\alpha}{\alpha+\gamma}$ and $p_{21} = 1$, we can define our conditioned waiting times according to the last section about semi-Markov processes:

$$\begin{aligned} \Psi_{01} &= \lambda e^{-\lambda t} & \Psi_{10} = \gamma e^{(\alpha+\gamma)t} \\ \Psi_{12} &= \alpha e^{-(\alpha+\gamma)t} & \Psi_{21} = \gamma e^{-\gamma t}. \end{aligned} \tag{17}$$

After Laplace transform we obtain:

$$\begin{aligned} \tilde{\Psi}_{01} &= \frac{\lambda}{\lambda + s} & \tilde{\Psi}_{10} = \frac{\gamma}{\alpha+\gamma+s} \\ \tilde{\Psi}_{12} &= \frac{\alpha}{\alpha+\gamma+s} & \tilde{\Psi}_{21} = \frac{\gamma}{\gamma+s}. \end{aligned} \tag{18}$$

We now solve for the vector of the fluxes $\tilde{\mathbf{I}}^j = (\tilde{I}^j_{01}, \tilde{I}^j_{10}, \tilde{I}^j_{12}, \tilde{I}^j_{21})$ the matrix equation 13. With the initial function vectors $\tilde{\mathbf{f}}^0 = (\tilde{\Psi}_{01}, 0, 0, 0)$ and $\tilde{\mathbf{f}}^0 = (0, 0, 0, \tilde{\Psi}_{21})$ and the matrix of conditioned waiting times

$$\tilde{\boldsymbol{\Psi}} = \begin{pmatrix} 0 & \tilde{\Psi}_{01} & 0 & 0 \\ \tilde{\Psi}_{10} & 0 & 0 & \tilde{\Psi}_{10} \\ \tilde{\Psi}_{12} & 0 & 0 & \tilde{\Psi}_{12} \\ 0 & 0 & \tilde{\Psi}_{21} & 0 \end{pmatrix}.$$

Next we plug these fluxes into the equation for the Laplace transformed FPT

density Eq. 14 calculated in the last section:

$$\tilde{F}_{0,2}(s) = \frac{\tilde{\Psi}_{01}\tilde{\Psi}_{12}}{1-\tilde{\Psi}_{01}\tilde{\Psi}_{10}} = \frac{\alpha\lambda}{\alpha(s+\lambda)+s(\gamma+s+\lambda)}. \tag{19}$$

The mean first passage time is given by

$$\langle t \rangle = -\frac{\partial}{\partial s}\tilde{F}_{i,j}(s)|_{s=0} = \frac{\alpha+\lambda+\gamma}{\alpha\lambda}. \tag{20}$$

Because the structure of the solution in terms of the Laplace transformed FPT density is quite simple for that minimal system, it is possible to use the inverse Laplace transform to obtain the FPT density directly:

$$F_{0,2}(t) = \alpha\lambda \frac{\left(e^{-\frac{1}{2}(Y-\frac{\lambda}{2}\sqrt{-4\alpha\lambda+Y^2})t} - e^{-\frac{1}{2}(Y+\frac{\lambda}{2}\sqrt{-4\alpha\lambda+Y^2})t}\right)}{\sqrt{-4\alpha\lambda+Y^2}}, \tag{21}$$

where $Y = \alpha+\gamma+\lambda$.

The result is a bi-exponential density. If we impose a time-scale separation, meaning $\alpha \ll \lambda$, the second exponential goes to zero for growing λ. This leads to a convergence to an exponential distribution of the FPT, as observed in the parameter studies for the stochastic calcium model.

4 Laplace transformation of the generalised exponential distribution

The generalised exponential (GE) distribution is a generalised form of the human mortality distribution discovered by Gompertz and Verhulst in the first half of the 19th century [7, 8]. For the analytic solution of the first passage time problem formulated in the main text, the Laplace transformation of the GE density function and its survival function are needed.

The GE density itself reads

$$\Psi_o(t) = \alpha\lambda\ (1-e^{-\lambda t})^{\alpha-1}\ e^{-\lambda t}. \tag{22}$$

We want to solve its Laplace transform whis is formally given by the following integral:

$$\mathcal{L}\{\Psi_o\}(s) = \int_0^\infty \alpha\lambda\ (1-e^{-\lambda t})^{\alpha-1}\ e^{-\lambda t}\ e^{-st}dt. \tag{23}$$

We start by substituting $y = e^{-t}$, which gives

$$\alpha\lambda \int_0^1 (1-y^\lambda)^{\alpha-1}\ y^{\lambda+s-1}dy. \tag{24}$$

Next we use the substitution $x = y^\lambda$:

$$\alpha \int_0^1 (1-x)^{\alpha-1}\ x^{\frac{s}{\lambda}} dx. \tag{25}$$

Now we use the definition of the beta function, written as an integral, it reads:

$$B(k,l) = \int_0^1 (1-x)^{k-1}\ x^{l-1} dx. \tag{26}$$

There is also a form involving the Euler Γ-function:

$$B(k,l) = \frac{\Gamma(k)\Gamma(l)}{\Gamma(k+l)}. \tag{27}$$

By using $l = \frac{s}{\lambda} + 1$ and substituting $k = \alpha$, we obtain from Eq. 25:

$$\mathcal{L}\{\Psi_o\}(s) = \frac{\alpha\Gamma(\alpha)\Gamma(\frac{s}{\lambda}+1)}{\Gamma(\alpha+\frac{s}{\lambda}+1)}. \tag{28}$$

The Laplace transformation for the survival function

$$\bar{\Psi}_o(t) = 1 - (1-e^{-\lambda t})^\alpha \tag{29}$$

can be done in analogy. This time we want to solve

$$\mathcal{L}\{\bar{\Psi}_o\}(s) = \frac{1}{s} - \int_0^\infty (1-e^{-\lambda t})^\alpha dt, \tag{30}$$

where we already used the trivial Laplace transformation of a constant. We substitute $y = e^{-t}$ followed by the second substitution $x = y^\lambda$ again to end up with

$$\frac{1}{s} - \frac{1}{\lambda}\int_0^1 (1-x)^\alpha\ x^{\frac{s}{\lambda}-1} dx. \tag{31}$$

Now we recall the beta function (Eq. 27) and finally substitute $l = \frac{s}{\lambda}$ and $k = \alpha + 1$ to obtain the solution:

$$\mathcal{L}\{\bar{\Psi}_o\}(s) = \frac{1}{s} - \frac{\Gamma(\alpha+1)\Gamma(\frac{s}{\lambda})}{\lambda\Gamma(\alpha+\frac{s}{\lambda}+1)}. \tag{32}$$

Note that the powers of $\bar{\Psi}_o$ needed for the construction of the conditioned waiting times can be written as

$$\bar{\Psi}_o^n = \sum_{k=0}^{\mathrm{n}} \binom{n}{k} (-1)^k (1-e^{-\lambda t})^{k\alpha}. \tag{33}$$

These are just sums of the original terms with a rescaled shape parameter $k\alpha$, and therefore their Laplace transforms are sums of the accordingly rescaled results given above in Eq. 32.

5 Details for the minimal mechanistic Ca^{2+} model

5.1 Closing probability $\psi_c(t)$.

The probability density of the waiting time until an IP_3R closes again, $\psi_c(t)$, can be derived from the experimental finding that the individual channels in a cluster close independently with closing rate γ, which does not depend on the Ca^{2+} concentration. It is a molecular property of IP_3R [5, 9]. Based on this, we can write the waiting time density for closing of an IP_3R cluster with, on average, N_{ch} channels involved in a puff:

$$\psi_c(t) = N_{ch}\gamma e^{-\gamma t}(1 - e^{-\gamma t})^{N_{ch}-1}. \tag{34}$$

The channel closing rate γ has recently been determined by total internal reflection fluorescence (TIRF) microscopy and is 17 s^{-1} in SH-SY5Y cells [9]. Equation 34 fits data from TIRF microscopy [9] and leads to Ca^{2+} spike statistics close to experimental data if incorporated into the hierarchic stochastic model [5].

The form of $\psi_c(t)$ exactly constitutes a GE distribution with parameters N_{ch} and γ substituting for α and λ from the formulation of the previous section. Using its result we readily write for the Laplace transform of the closing probability:

$$\mathcal{L}\{\Psi_c\}(s) = \frac{N_{ch}\Gamma(N_{ch})\Gamma(\frac{s}{\gamma}+1)}{\Gamma(N_{ch}+\frac{s}{\gamma}+1)}. \tag{35}$$

However, in contrast to the general GE distribution, the parameter N_{ch} takes only postive integers. This allows for a simpler expression, i.e. by using the binomial theorem we can write:

$$\Psi_c(t) = N_{ch}\gamma e^{-\gamma t} \sum_{k=0}^{N_{ch}-1} \binom{N_{ch}-1}{k} (-1)^k e^{-k\gamma t}, \tag{36}$$

where we dropped $1^{N_{ch}-1-k} = 1$ inside the sum. After multiplication we get:

$$\Psi_c(t) = N_{ch}\gamma \sum_{k=0}^{N_{ch}-1} \binom{N_{ch}-1}{k} (-1)^k e^{-(k+1)\gamma t}. \tag{37}$$

With this we just have to Laplace transform a sum of exponentials, and we finally obtain:

$$\tilde{\Psi}_c(s) = \mathrm{N_{ch}}\gamma \sum_{k=0}^{\mathrm{N_{ch}}-1} \binom{\mathrm{N_{ch}}-1}{k} (-1)^k \frac{(-1)^k}{s+(k+1)\gamma}. \tag{38}$$

This form of the Laplace transformed closing time can be used for construction of the conditioned waiting times (see below), which are needed for computation of the Ca^{2+} spiking statistics by Eq. 15

5.2 Detailed derivation of the opening probability densities $\psi_o(t)$

The analytical approximations of the model dependencies on cellular parameters (Section Methods of the main text) are based on computations of the opening probability densities $\psi_{o,i}(t)$ by a method developed in an earlier study by the Falcke lab [10, 11]. The method uses the De Young-Keizer model [12] for the description of the individual IP_3Rs with the parameter values given in Tab. S1. On the basis of that model, ψ_o can be computed from the master equation describing the random channel state changes. Briefly, the De Young-Keizer model assumes that a channel is open when three out of the four subunits of the IP_3R are bound by IP_3 and activating Ca^{2+}, but not by inhibiting Ca^{2+}. The transition rates between the states could be determined by experiments to some extent.

By those computations, we found that the dependencies of the $\psi_{o,i}(t)$ on cellular parameters are well approximated by analytical functions of the parameters α and λ of GE distributions (see Fig. 2 of the main text):

$$\alpha(x) = \frac{V_x x^{n_x}}{(K_x)^{n_x} + x^{n_x}} + 1 \tag{39}$$

$$\lambda(x) = \frac{U_x x^{m_x}}{(L_x)^{m_x} + x^{m_x}}, \tag{40}$$

where $x = [Ca^{2+}]$, $[IP_3]$, $\mathrm{N_{ch}}$. The first row in Tab. S2 shows the fitting parameters for the dependence on the Ca^{2+} concentration. Note that Eq. 40 is almost linear in this case (see Fig. 2), so that $\lambda([Ca^{2+}]) \approx 62.88\ \mu M^{-1}s^{-1} \times [Ca^{2+}] - 13.56\ s^{-1}$.

To determine the other parameters, we started by the random opening of only one single cluster (a puff), which is always the first step in the stochastic process generating a global Ca^{2+} spike. These openings can be modelled by an exponential distribution with puff rate λ_0 [5, 13], so that λ_0 is determined by Eq. 40 only. Based on recent experimental data [13], we chose a puff rate $\lambda_0 =$

0.31 s^{-1} (see main text) at standard parameter values (N_{ch}=5, [IP_3]=1 μM), which corresponds to a base-level Ca^{2+} concentration $c_0 = 0.12$ μM in the De Young-Keizer-model. Based on that, we determined the dependencies of λ_0 on N_{ch} and [IP_3] (rows with 'open clusters=0' in Tab. S2). We found that the relation of λ_0 and N_{ch} is linear, so that $K_{N_{ch}} = n_{N_{ch}} = 0$.

The opening probabilities in the vicinity of already open clusters nearby are computed at higher Ca^{2+} concentrations. The needed values of [Ca^{2+}] are computed from an appropriate Ca^{2+} diffusion profile as in [5]. The Ca^{2+} concentration depends on the number of open clusters N_o:

$$[\mathrm{Ca}^{2+}] = c_0 + N_o \times c_1, \tag{41}$$

where $c_1 = 0.426$ μM is the Ca^{2+} concentration resulting from one nearby open cluster. The simple form of Eq. 41 results from the tetrahedral geometry and from the assumption of free boundaries [5]. It can be replaced by a diffusion problem taking more cellular details into account without difficulty. Based on Eq. 41, we obtain all fitting parameters needed to reproduce the minimal stochastic Ca^{2+} model analysed in the main text (Tab. S2).

5.3 Implementation of the tetrahedron model.

If we consider four Ca^{2+} channel clusters forming the edges of a tetrahedron, all states with the same number of open clusters are equivalent with respect to the resulting Ca^{2+} concentration profile. The system therefore is a linear chain with $N = K+1 = 5$ distinct states, corresponding to $(0,1,2,3,4)$ open clusters. The probability not to have left a state by time t after arrival at time 0 is $\bar{\psi}_{o,c} = 1 - \int_0^t \psi_{o,c}(t')dt'$, for opening or closing of a cluster, respectively. The waiting time distributions depend on [Ca^{2+}] and consequently on the system state. We therefore add subscript i, i.e. $\psi_{o,i}$ which describes the opening probability of a cluster at system state i:

$$\begin{aligned} \Psi_{i,i+1} &= (4-i)\psi_{o,i} \times \left(\bar{\psi}_c\right)^i \times \left(\bar{\psi}_{o,i}\right)^{3-i} \\ \Psi_{i,i-1} &= i\psi_c \times \left(\bar{\psi}_c\right)^{i-1} \times \left(\bar{\psi}_{o,i}\right)^{4-i}, \end{aligned} \tag{42}$$

where the prefactors account for the multiplicity of the transitions. For a linear chain with $N = 5$ possible system states, the Laplace transformed FPT density is given by Eq. 15. Thus, it is necessary to find the Laplace transforms of the products of all individual transitions $\psi_o, \psi_c, \bar{\psi}_o, \bar{\psi}_c$. The waiting time density for the opening of the first cluster (Ψ_{01}) is a pure exponential and the density for the closing events can be written as a sum of exponentials (see Eq. 37). The

Laplace transform of exponential functions is simply $\mathcal{L}\{\lambda_0 e^{-\lambda_0 t}\} = \lambda_0/(\lambda_0 + s)$. The slightly more elaborate Laplace transformation for the GE-distribution can be found above in the SI section 4.

6 Detailed derivation of the generic model

The foundation of the generic model comes from the strong time scale separation between the average time between single puffs, and the average time needed for the opening of further clusters. With standard parameters the $S_0 \to S_1$ transition occurs on average every 7.8 s, whereas the $S_1 \to S_2$ transition lasts on average 0.035 seconds. The transition back to the ground state $S_1 \to S_0$ also occurs rather fast with 0.15 s. Compared to the average ISI ($\sim$ 250 s) the system decides almost instantaneously if a single puff becomes a global spike, or if it relaxes back to the ground state. The probability to reach state S_4 starting in S_1 before going back to S_0 is the splitting probability C_{14} (see also main text). Knowing this, we separated the whole stochastic process generating the global Ca^{2+} spikes into one inhomogeneous Poisson process describing the puffs, and a Bernoulli trial with success probability C_{14}. For the analytically tractable case, the puff process was a homogeneous Poisson process with a constant puff rate λ_0. We now include a negative feedback to capture phenomena like Ca^{2+} store depletion. So we recast the puff process in the following form

$$\begin{aligned} \lambda(t) &= \lambda_0(1 - e^{-\xi t}) \\ p_p(t) &= \lambda(t) e^{-\Lambda(t)}, \end{aligned} \tag{43}$$

with $\lambda(t)$ as time dependent puff rate tending to the asymptotic rate λ_0 and the intensity $\Lambda(t) = \int_0^t \lambda(\tau) d\tau$

One can now ask for the probability that at a time point t a puff occurs and triggers a global Ca^{2+} spike. Conditioned on the ground state S_0 we are looking

for the time for the first Ca^{2+} spike to occur, the ISI. Formally this leads to:

$$
\begin{aligned}
p_s(t) &= C_{14}\lambda(t) \times \Big(e^{-\Lambda(t)} \\
&\quad +(1-C_{14})\int_0^t e^{-\Lambda(t_1)}\lambda(t_1) \\
&\quad +(1-C_{14})^2\int_0^t\int_{t_1}^t e^{-\Lambda(t_1)}\lambda(t_1)e^{-(\Lambda(t_2)-\Lambda(t_1))}\lambda(t_2)e^{-(\Lambda(t)-\Lambda(t_2))}dt_1dt_2 \\
&\quad + ...\Big) \\
&= C_{14}\lambda(t)e^{-\Lambda(t)}\Big(1+(1-C_{14})\int_0^t\lambda(t_1)dt_1 \\
&\quad + (1-C_{14})^2\int_0^t\int_{t_1}^t\lambda(t_1)\lambda(t_21)dt_1dt_2 + ...\Big) \qquad (44)
\end{aligned}
$$

This expression contains the probabilities for all possible puffs occurring before t not leading to a spike, the failed Bernoulli trials with probability $(1-C_{14})$. The very first term is the probability that at t a puff occurs and becomes a global spike and no other puff occurs in $[0,t)$, the second term is the probability for one failed puff in $[0,t)$, the third term handles two failed puffs and so on. Following permutation and symmetry arguments outlined in the book by Van Kampen [14] we may write:

$$
\begin{aligned}
p_s(t) &= C_{14}\lambda(t)e^{-\Lambda(t)}\Big(\sum_{n=0}^{\infty}\frac{1}{n!}\big((1-C_{14})\Lambda(t)\big)^n\Big) \\
&= C_{14}\lambda(t)e^{-C_{14}\Lambda(t)} = \kappa(t)e^{-C_{14}\Lambda(t)}. \qquad (45)
\end{aligned}
$$

The resulting stochastic process for the Ca^{2+} spiking is also a inhomogeneous Poisson process with the spike rate $\kappa(t) = C_{14}\lambda(t)$. The new rate is just the puff rate refined with the splitting probability C_{14}, this property is known as Poisson splitting. It may be generalised to heterogeneous puff sites with individual splitting probabilities $C_{1N,i}$ and puff rates $\lambda_{0,i}$, see also main text.

7 Error analysis for the generic model

The generic model can only be an approximation for the real stochastic process governing the Ca^{2+} spike generation. The applied Poisson splitting completely neglects the dynamics of the system between the individual states, instead it implicitly sets all transition times except for the $S_0 \to S_1$ transition to zero. So we expect to underestimate the true average ISI, $T_{av}^{gm} < T_{av}^{tr}$. The time the system spends in processing the failed puffs is a major source for the under-

estimation. The number of failed puffs is on average given by $1/C_{14}$, which is for standard parameters $\sim$ 100. To exactly quantify the approximation error of the generic model we compared its results for $\xi \rightarrow \infty$ (no feedback) with the analytic results given by the hierarchic stochastic model. As expected the error grows linear with $1/C_{14}$ (Fig. S1A). We also computed the relative error $(\mathrm{T}_{\mathrm{av}}^{\mathrm{tr}} - \mathrm{T}_{\mathrm{av}}^{\mathrm{gm}})/\mathrm{T}_{\mathrm{av}}^{\mathrm{tr}}$ as function of IP_3 , and state that it approaches zero for $\mathrm{IP}_3 \rightarrow 0$ and saturates for $\mathrm{IP}_3 \rightarrow \infty$ (Fig. S1B). Our main argument for the generic model is the time scale separation between puff and spike events. In accordance with that the approximation error gets bigger with weaker time scale separation, realised by a smaller channel closing rate γ (Fig. S1A-B).

References

[1] Cinlar E (1975) Markov Renewal Theory: A survay. Management Science 21.

[2] Schlicht R, Winkler G (2008) A delay stochastic process with applications in molecular biology. J Math Biol 57: 613-648.

[3] Cox D (1970) Renewal Theory. Methuen & Co.

[4] Prager T, Falcke M, Schimansky-Geier L, Zaks MA (2007) Non-Markovian approach to globally coupled excitable systems. Phys Rev E 76: 011118.

[5] Thurley K, Falcke M (2011) Derivation of Ca^{2+} signals from puff properties reveals that pathway function is robust against cell variability but sensitive for control. Proc Natl Acad Sci U S A 108: 427–32.

[6] Gillespie DT (1992) Markov Processes - An Introduction for Physical Scientists. Academic Press.

[7] Gupta RD, Kundu D (2007) Generalized exponential distribution: Existing results and some recent developments. J Statist Plann Inference 137.

[8] Gompertz B (1825) On the Nature of the Function Expressive of the Law of Human Mortality, and on a New Mode of Determining the Value of Life Contingencies. Phil Trans Royal Soc 115.

[9] Smith IF, Parker I (2009) Imaging the quantal substructure of single IP3R channel activity during Ca^{2+} puffs in intact mammalian cells. Proc Natl Acad Sci U S A 106: 6404-9.

[10] Higgins ER, Schmidle H, Falcke M (2009) Waiting time distributions for clusters of IP3 receptors. J Theor Biol 259: 338-349.

[11] Thul R, Thurley K, Falcke M (2009) Toward a predictive model of Ca^{2+} puffs. Chaos 19: 037108.

[12] De Young GW, Keizer J (1992) A single-pool inositol 1,4,5-trisphosphate-receptor-based model for agonist-stimulated oscillations in Ca^{2+} concentration. Proc Natl Acad Sci U S A 89: 9895-9.

[13] Thurley K, Smith IF, Tovey SC, Taylor CW, Parker I, et al. (2011) Timescales of IP(3)-Evoked Ca(2+) Spikes Emerge from Ca(2+) Puffs Only at the Cellular Level. Biophys J 101: 2638-44.

[14] Van Kampen NG (2002) Stochastic Processes in Physics and Chemistry. Amsterdam: Elsevier Science B.V.
